# Supplementary material for: Young’s Experiment with Entangled Bipartite Systems: The Role of Underlying Quantum Velocity Fields
Source: Entropy (Basel). 2023 Jul 17;25(7):1077. doi: 10.3390/e25071077 (PMC10378373; doi:10.3390/e25071077)
Supplement: Supplementary file 1 [file entropy-25-01077-s001.zip › entropy-2482444-supplementary.pdf]

Article

Young's experiment with entangled bipartite systems: The role of underlying quantum velocity fields – Supplementary Material –

Ángel S. Sanz

Department of Optics, Faculty of Physical Sciences, Universidad Complutense de Madrid,
Pza. Ciencias 1, Ciudad Universitaria, 28040 Madrid, Spain
* Correspondence: a.s.sanz@fis.ucm.es; Tel.: +34-91-394-4673

Version June 19, 2023 submitted to Entropy

The figures below show with more detail the dispersive effect led by the velocity fields $v^X(x, y|t)$ and $v^Y(x, y|t)$ over swarms of 441 initial conditions distributed in an evenly-spaced 21 × 21 square array covering each Gaussian distribution and centered at the Gaussian centroid. Moreover, the full three-dimensional trajectories corresponding to the cases analyzed in Figure 6 (main article) are shown in Figure S5 for a better understanding of the dynamics exhibited in the joint (*XY*) three-dimensional configuration space.

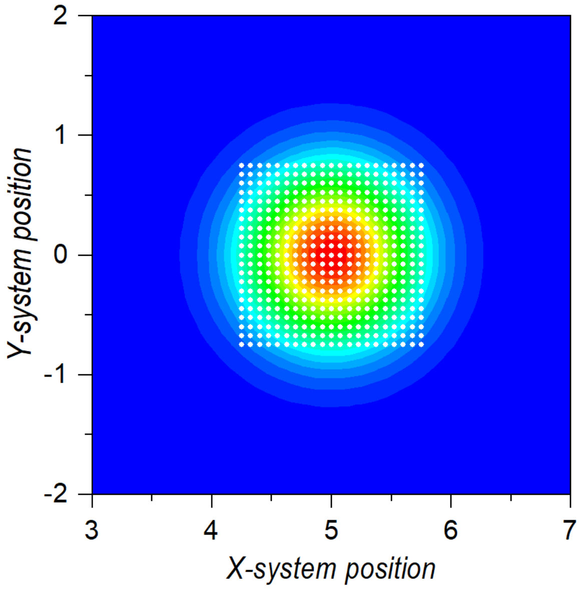

Figure S1. Evenly-spaced 21 × 21 squared array used to specify the initial conditions (solid white circles) of a set of 441 Bohmian trajectories. As an example, a single Gaussian distribution is shown, although the same array will be considered for all Gaussian distributions involved in each state analyzed. In this particular instance, the Gaussian distribution corresponds to the right-hand side wave packet of the *X*-subsystem superposition coupled to the *Y*-subsystem single Gaussian wave packet. The numerical values used here are: $\sigma_0 = 0.5$, $p_0 = 0$, $m = 1$, and $\hbar = 1$.

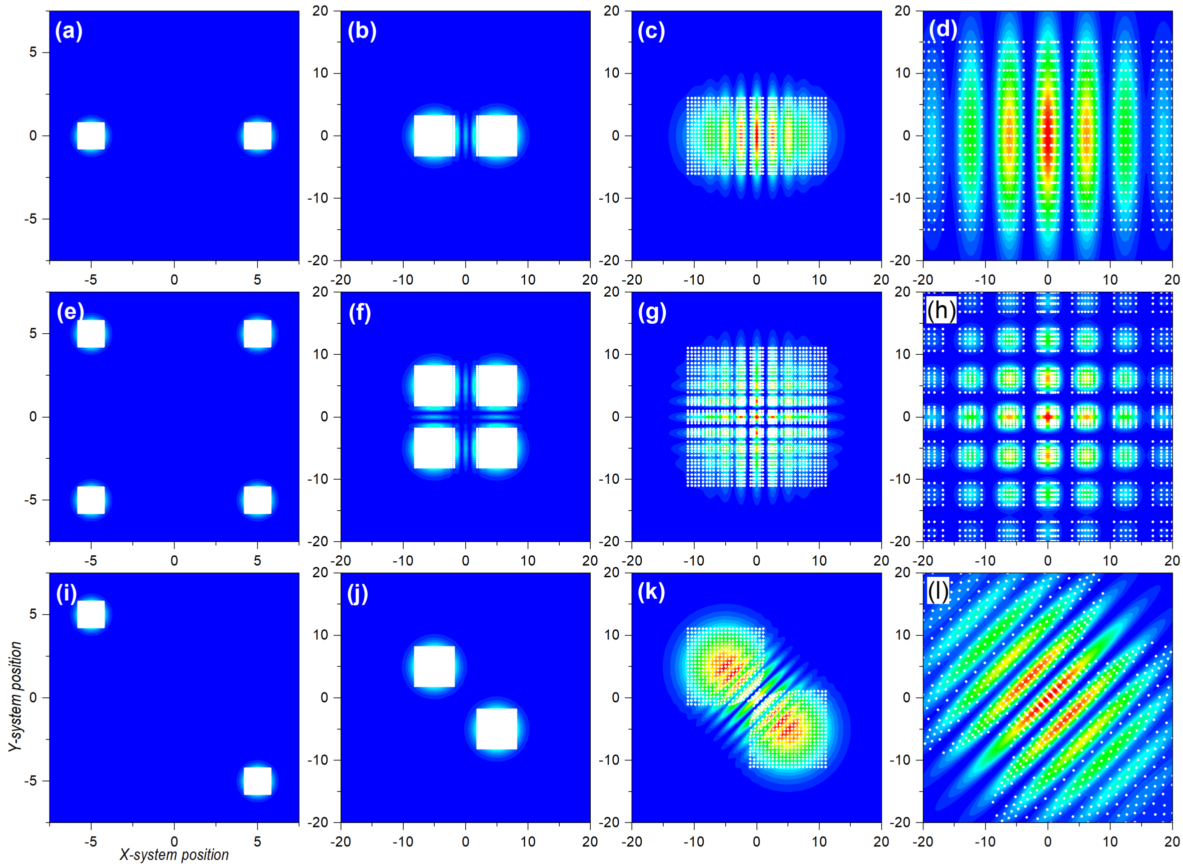

Figure S**2.** Contour plots illustrating several stages of the evolution of the probability density for three bipartite systems. (a-d) Upper row: Uncorrelated bipartite state described by a two-Gaussian superposition for X and single Gaussian for Y. (e-h) Central row: Uncorrelated bipartite state described by a two-Gaussian superposition for both X and Y. (i-l) Lower row: Entangled bipartite state described by a Bell-type state. From left to right: $t = 0$, $t = 2$, $t = 4$, and $t = 10$. In the first column panels, the sets of 441 markers (solid white circles) distributed in squared arrays superimposed to each Gaussian distribution denote the swarms of initial conditions considered in the calculation of Bohmian trajectories. The markers in the next column panels show evidence of the difussion process that is taking place, which strongly depends on the different dyanmics generated by each joint state. The numerical values used in the simulations are: $x_0 = 0$ for the single Gaussian and $d = 10$ for other cases with two Gaussians, $\sigma_0 = 0.5$, $p_0 = 0$, $m = 1$, and $\hbar = 1$.

© 2023 by the authors. Submitted to *Entropy* for possible open access publication under the terms and conditions of the Creative Commons Attribution (CC BY) license (http://creativecommons.org/licenses/by/4.0/).

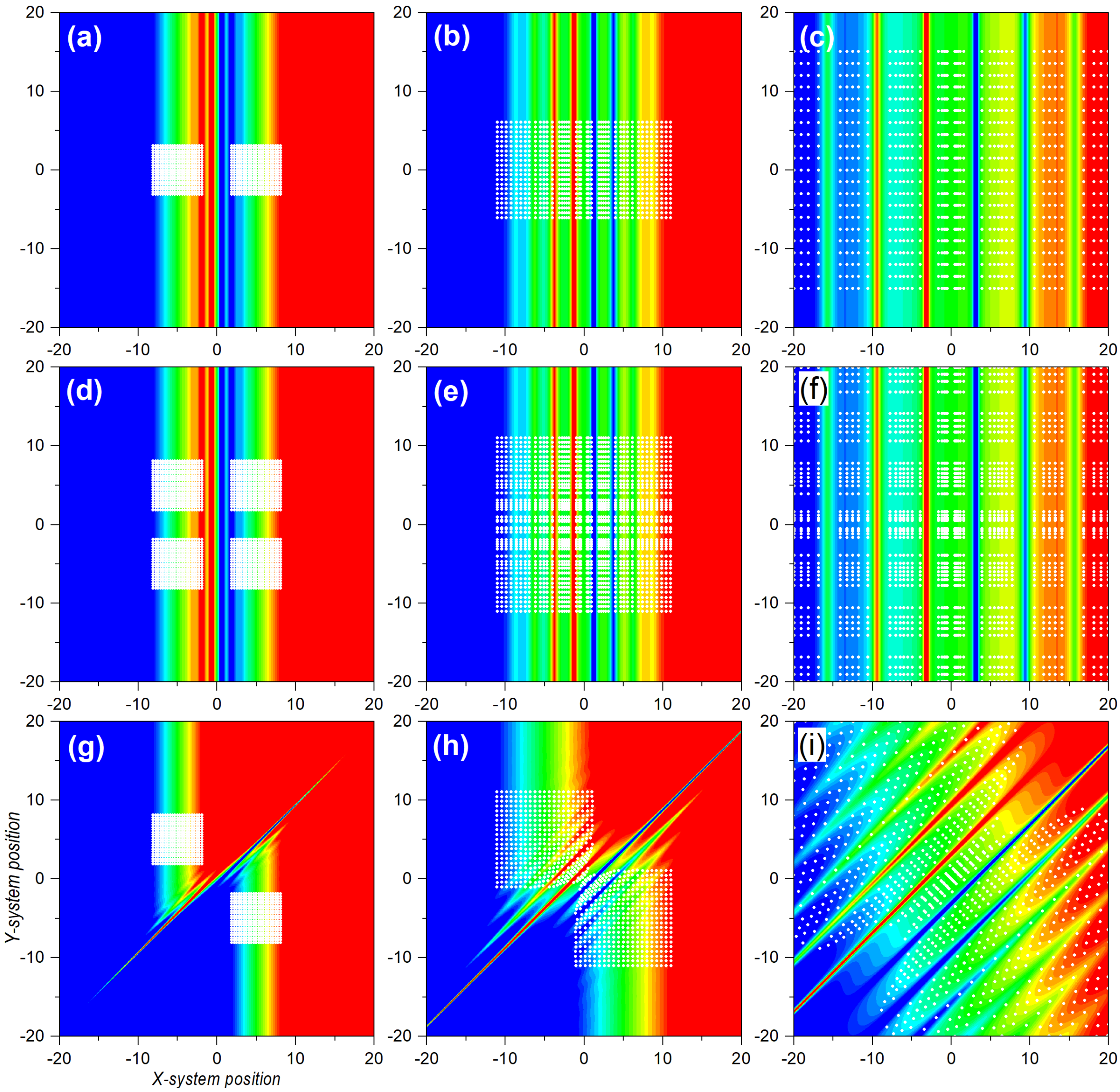

Figure S**3.** Contour plots illustrating several stages of the evolution of the x-component of the transverse velocity field, $v^X(x, y|t)$, for the three bipartite systems of Figure S2. (a-c) Upper row: Uncorrelated bipartite state described by a two-Gaussian superposition for X and single Gaussian for Y. (d-f) Central row: Uncorrelated bipartite state described by a two-Gaussian superposition for both X and Y. (g-i) Lower row: Entangled bipartite state described by a Bell-type state. From left to right: $t = 2$, $t = 4$, and $t = 10$. The markers show how the velocity field directed along the x-direction is affecting the diffusion process of the trajectories in that direction. The numerical values used in the simulations are: $x_0 = 0$ for the single Gaussian and $d = 10$ for other cases with two Gaussians, $\sigma_0 = 0.5$, $p_0 = 0$, $m = 1$, and $\hbar = 1$.

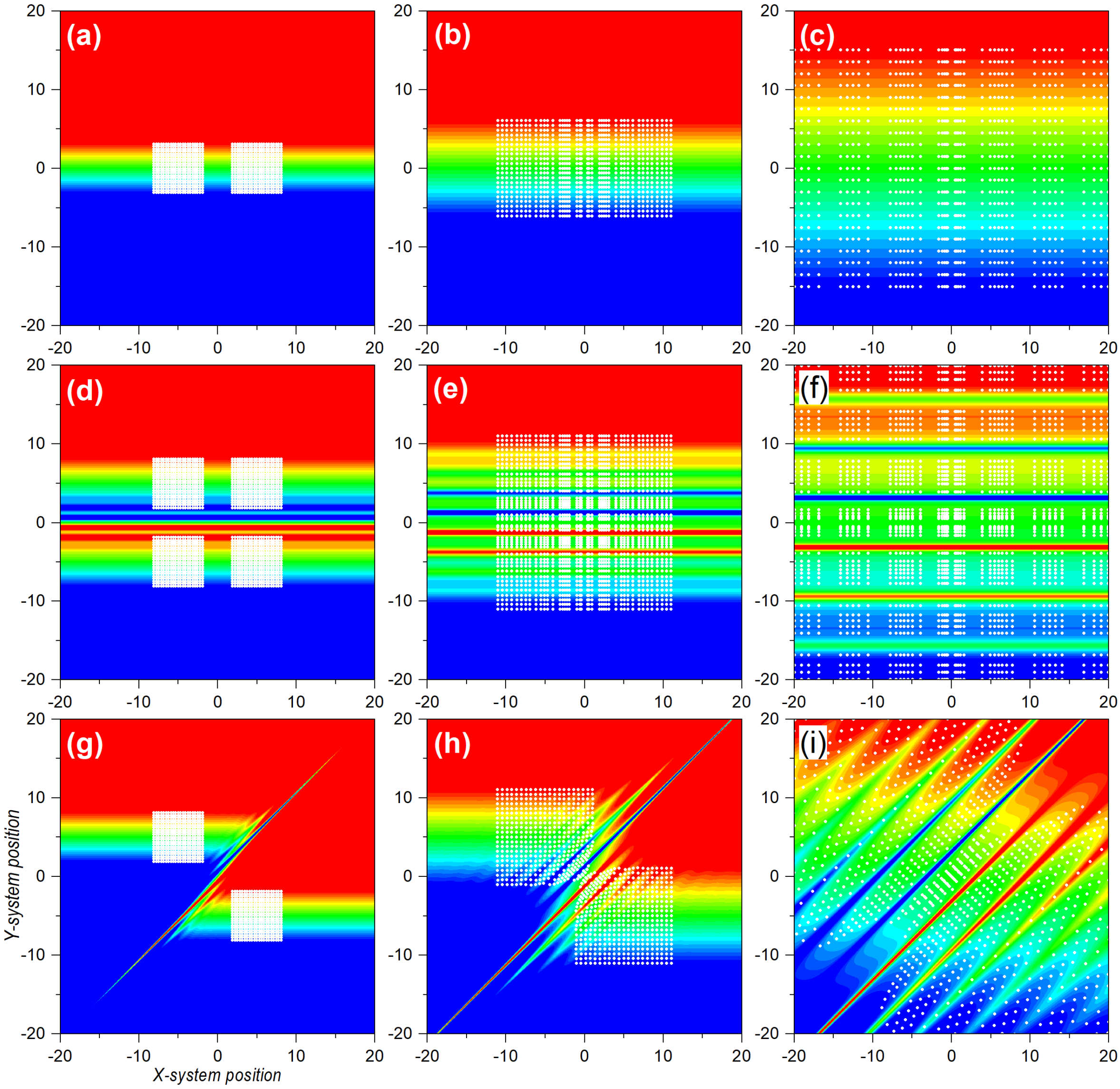

Figure S4. Contour plots illustrating several stages of the evolution of the y-component of the transverse velocity field, $v^Y(x, y|t)$, for the three bipartite systems of Figure S2. (a-c) Upper row: Uncorrelated bipartite state described by a two-Gaussian superposition for X and single Gaussian for Y. (d-f) Central row: Uncorrelated bipartite state described by a two-Gaussian superposition for both X and Y. (g-i) Lower row: Entangled bipartite state described by a Bell-type state. From left to right: $t = 2$, $t = 4$, and $t = 10$. The markers show how the velocity field directed along the y-direction is affecting the diffusion process of the trajectories in that direction. The numerical values used in the simulations are: $x_0 = 0$ for the single Gaussian and $d = 10$ for other cases with two Gaussians, $\sigma_0 = 0.5$, $p_0 = 0$, $m = 1$, and $\hbar = 1$.

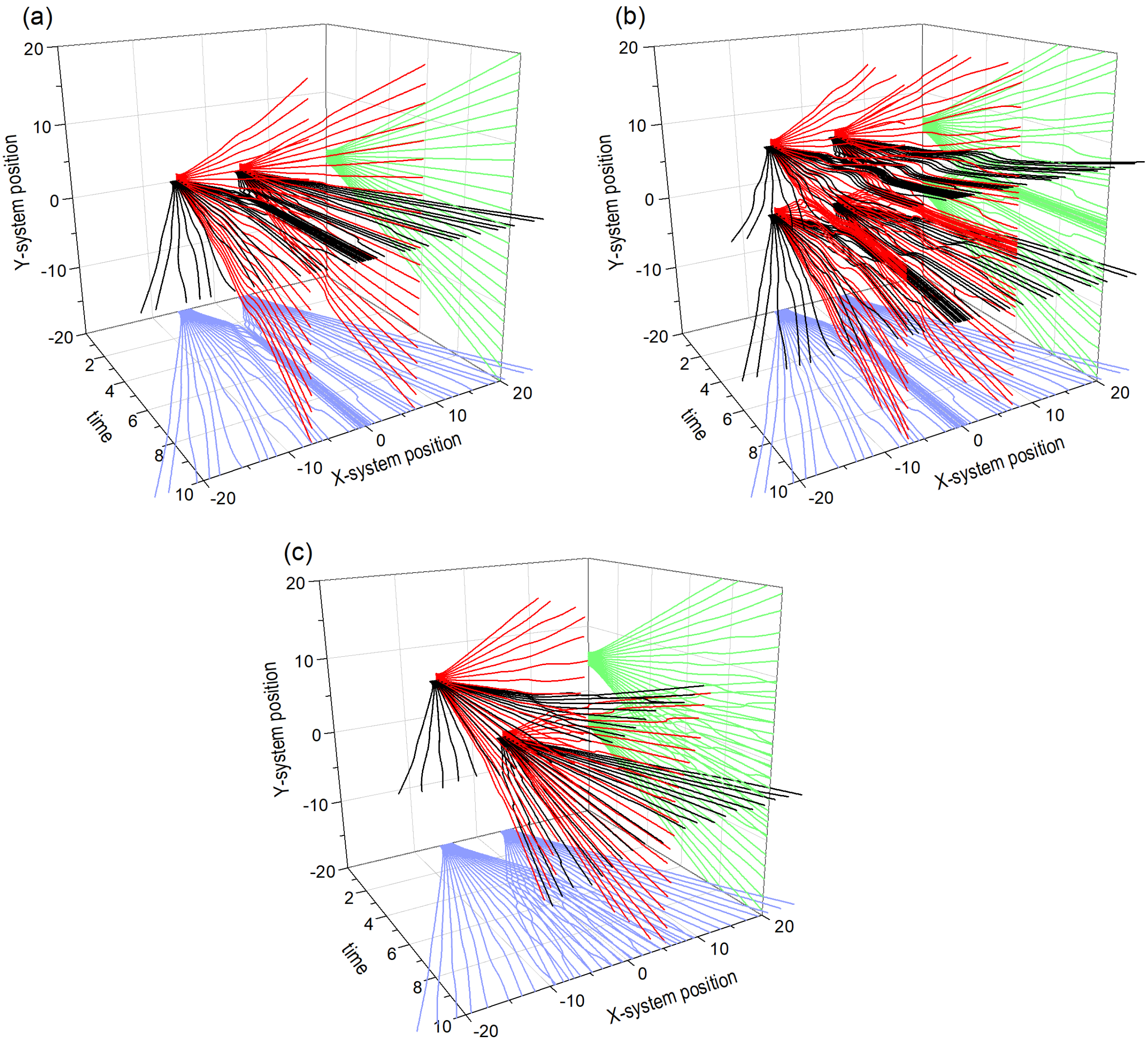

Figure S5. Three-dimensional representation of the Bohmian trajectories showed in Fig. 6 (main article), which illustrate the dynamics associated with the three bipartite systems of Fig. 3 (main article): (a) uncorrelated bipartite state described by a two-Gaussian superposition for X and single Gaussian for Y; (b) the trajectories for each subsystem are plotted in the top and bottom panels, respectively; and (c) entangled bipartite state described by a Bell-type state. As it is shown in the corresponding first column panels in Fig. 3 (main article), the initial conditions have been chosen considering 21 equidistant positions covering the Gaussian wave packet (along the x or the y directions, depending on whether we are interested in the X or the Y subsystem, respectively). In all plots, the x-component of the trajectories is represented with solid black line, and the y-component with solid red line. The projections onto the bottom and side correspond to dynamics exhibited in the corresponding X and Y subspaces. The numerical values used in the simulations are: $x_0 = 0$ for the single Gaussian and $d = 10$ for other cases with two Gaussians, $\sigma_0 = 0.5$, $p_0 = 0$, $m = 1$, and $\hbar = 1$.
